# Underestimation of the number of hidden objects

**DOI:** 10.1167/jov.23.2.1

**Published:** 2023-02-01

**Authors:** Hui Men, Anna Altin, Alexander C. Schütz

**Affiliations:** 1 Allgemeine und Biologische Psychologie, Philipps-Universität Marburg, Marburg, Germany; 2 Center for Mind, Brain and Behaviour, Philipps-Universität Marburg, Marburg, Germany

**Keywords:** numerosity, perceptual completion, occlusion, confidence

## Abstract

The perceptual representation of our environment does not only involve what we actually can see, but also inferences about what is hidden from our sight. For example, in amodal completion, simple contours or surfaces are filled-in behind occluding objects allowing for a complete representation. This is important for many everyday tasks, such as visual search, foraging, and object handling. Although there is support for completion of simple patterns from behavioral and neurophysiological studies, it is unclear if these mechanisms extend to complex, irregular patterns. Here, we show that the number of hidden objects on partially occluded surfaces is underestimated. Observers did not consider accurately the number of visible objects and the proportion of occlusion to infer the number of hidden objects, although these quantities were perceived accurately and reliably. However, visible objects were not simply ignored: estimations of hidden objects increased when the visible objects formed a line across the occluder and decreased when the visible objects formed a line outside of the occluder. Confidence ratings for numerosity estimation were similar for fully visible and partially occluded surfaces. These results suggest that perceptual inferences about what is hidden in our environment can be very inaccurate und underestimate the complexity of the environment.

## Introduction

In our cluttered environment, it often happens that objects are partially or completely hidden by other objects (Nanay, 2018). However, the perceptual representation of those partially or fully occluded objects is important for survival in the natural world: missing a predator can be deadly and missing patches of high-density of fruits leads to inefficient foraging.

Although visibility is reduced in the condition of occlusion, the lack of visual information can be compensated by our visual system via a constructive process of perception, namely, perceptual completion (Pessoa, Thompson, & Noë, 1998; Scherzer & Faul, 2019; Weil & Rees, 2011). In amodal completion (Michotte, Thinès, & Crabbé, 1964; Øhrn, Svalebjørg, Andersen, Ring, & Ekroll, 2019; Scherzer & Faul, 2019), partially occluded contours, surfaces or patterns are perceptually filled-in behind occluders to allow for a complete representation of the objects. Similar perceptual completion occurs in sensory gaps that are caused by the properties of the visual system, such as the blind-spot (Ramachandran, 1992) or the foveal rod scotoma (Gloriani & Schütz, 2019). Behavioral studies have extensively investigated according to which rules lines and contours are completed across sensory gaps and occluders (van Lier, van der Helm, & Leeuwenberg, 1995). Neurophysiological studies have shown that neurons in early visual cortex actively represent the completed information (de Weerd, 2006; Komatsu, 2006; Thielen, Bosch, van Leeuwen, van Gerven, & van Lier, 2019).

So far, the research on perceptual completion has focused on simple objects, leaving the complexity and the limitations of inferences about hidden information largely unexplored. Humans are able to extract efficiently the overall statistics of large visible areas and groups of redundant objects (Whitney & Yamanashi Leib, 2018), such as the average orientation (Dakin & Watt, 1997) or color (Webster, Kay, & Webster, 2014) of the objects in a set. Hence, one could expect that humans should be able to transfer and extrapolate those summary statistics easily to areas that are hidden from plain sight by other objects in the foreground.

In this study, we investigated how complex, sparse, and irregular patterns are completed behind an occluder, by probing the perceived number of visible and hidden objects. Our results showed that whereas the number of visible objects was perceived accurately and reliably, the number of hidden objects was strongly underestimated. This indicates that perceptual inferences about occluded objects underestimate the complexity of the environment and that the subjective impression of a complete and rich representation of our visual environment is illusory.

## Methods

### Design

**Figure 1.**
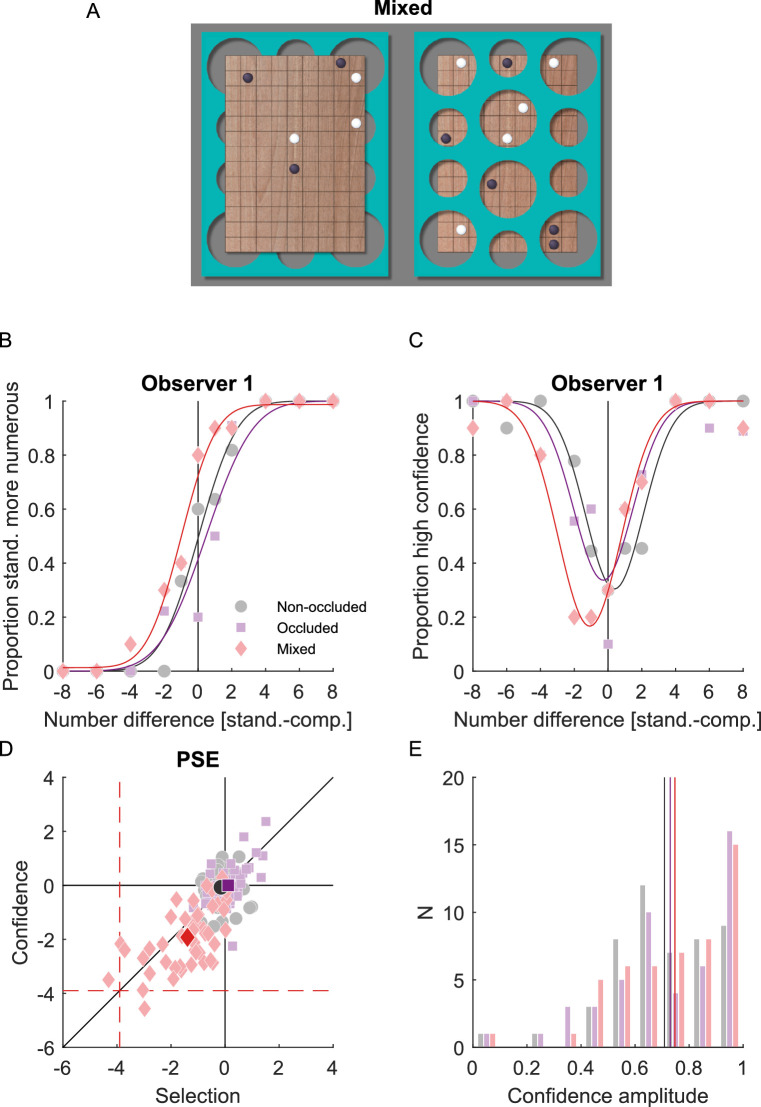
Discrimination of numerosity and visual confidence (experiment 1). (**A**) Stimulus in the mixed condition, where a non-occluded game board and an occluded game board were displayed simultaneously and had to be compared. (**B**) Psychometric function for the selection task of one representative observer. Proportion of standard stimulus more numerous is shown as a function of the difference in number between the standard and the comparison stimulus. (**C**) Psychometric function for the confidence task of one representative observer. Proportion of high confidence responses is shown as a function of the difference in number between the standard and the comparison stimulus. (**D**) Points of subjective equality (PSE) in the selection and the confidence task. Light colors represent individual observers; saturated colors the mean across observers. Error bars represent 95% confidence intervals. The black diagonal represents values with equal effects in the selection and the confidence task. Black horizontal and vertical lines represent values where occlusion is not taken into account. Dashed red lines represent values where occlusion is completely taken into account according to Equation 4. (**E**) Histogram of the amplitude modulation of the confidence. Thin vertical lines indicate the mean across observers.

In five experiments, we investigated the perception of hidden objects. In experiments 1 to 4, the visual scene consisted of a game board with different numbers of game pieces and a mesh that could act as an occluder (Figure 1A). In experiment 1, numerosity discrimination judgments and confidence judgments were collected under conditions with and without occlusion. In experiment 2, direct estimations of the number of visible and hidden objects were measured. In experiment 3, the perceived amount of occlusion was measured. Experiment 4 investigated how the arrangement of the visible pieces influences the estimation of the number of hidden pieces. In experiment 5, the visual scene consisted of a night sky with stars and clouds to test a more naturalistic context.

### Observers

We recruited observers from https://www.prolific.co/. All observers had normal or corrected-to-normal vision and provided written informed consent. All observers were naïve to the purpose of the experiment and were paid for their participation. The experiments were conducted in accordance with the Declaration of Helsinki (1964) and approved by the local ethics committee of the Psychology Department at Marburg University (proposal number 2020-43k).

In experiment 1, 49 observers (24 women and 25 men, mean age = 34 years, range = 19–45) took part. However, the data of five observers was incomplete due to technical problems. We excluded the incomplete data sets, resulting in a sample size of 44.

In experiment 2, 30 observers (18 women and 12 men, mean age = 34 years, range = 20–45) took part.

In experiment 3, 30 observers (20 women and 10 men, mean age = 33 years, range = 19–43) took part. Data from one observer was excluded, because she/he misunderstood the task instructions and reported the perceived occluded area in the non-occluded condition to be 1. The data of another observer was incomplete and therefore also excluded. This resulted in a sample size of 28.

In experiment 4, 31 observers (15 women and 16 men, mean age =33 years, range = 20–43) took part. The data of one observer was incomplete and therefore excluded. This resulted in a sample size of 30.

In experiment 5, 30 observers (17 women and 13 men, mean age = 33 years, range = 20–45) took part. The data of one observer was incomplete and therefore excluded. The data of another observer was excluded because they switched the estimation strategy during the experiment; estimations of the number of hidden stars were almost always 0 in the first 98 trials and larger than zero in the following trials. This resulted in a sample size of 28.

### Apparatus and stimuli

We used jsPsych (de Leeuw, 2015) to design the interface. The size of the stimuli was fixed relative to the resolution of the screen. Stimuli were displayed on a grey background (R = G = B = 128).

In experiments 1 to 4, the scene consisted of a game board with black and white game pieces and a mesh as an occluder (see Figure 1A). The average luminance over the black and white pieces, the luminance of the occluder, and the game board were matched. We first made the game board (luminance = 150) slightly brighter than the grey background. Then the color of the occluder was set to R = 100, G = 172, and B = 174, resulting in a luminance of 150 (0.3R + 0.59G + 0.11B; Recommendation ITU-R BT.601). The luminance of the plain black and white pieces was then set to be 70 (R = 68, G = 68, and B = 92) and 230 (R = 226, G = 226, and B = 255), resulting in an average luminance of 150. The occluders and game boards in all four experiments had shading effects, where the light was coming from the top-left corner to facilitate the impression of occlusion. The game pieces had consistent high-lights and shading effects. After adding the high-light effect to the pieces, the overall luminance of the black and white pieces was further adjusted to be 70 and 230 by slightly changing the values in three channels. Black and white game pieces were arranged randomly on the cells of the game board. The occurrence of the black and white pieces was balanced in each trial. The size of the occluder was 280 × 407 pixels (width × height). The size of the game board was 390 × 507 pixels (width × height), containing 117 cells with 13 rows × nine columns. The size of game pieces was 21 pixels.

In experiments 1, 2, and 3, each occluder had two patterns such that there would not be too many repeated occluders between adjacent trials. For each occluder, there were 12 holes, including six small ones and six large ones. The size of the occluder (*s*, *l*) was determined by the diameter of the holes, where *s* and *l* denote the diameter of the small and large holes in pixels, respectively. The holes in the occluder were arranged to be symmetric.

In experiment 1, there was only one size of occluder (40, 60 pixels).

In experiment 2, there were two sizes of occluders: small (40, 60 pixels) and large (30, 45 pixels).

In experiment 3, there were seven sizes of occluders, denoting seven proportions of occlusion: 0.25 (25, 35 pixels), 0.36 (30, 45 pixels), 0.43 (35, 50 pixels), 0.50 (39, 56 pixels), 0.58 (40, 60 pixels), 0.65 (54, 60 pixels), and 0.76 (58, 63 pixels). When scaling the stimuli to three scales (1, 0.75, and 0.5 times the original size of the stimuli), the occluder, the game board, and the game pieces were scaled simultaneously.

In experiment 4, the occluder consisted of two vertical (for numerosities of 5, 10, and 12) or horizontal (for numerosities of 7 and 14) bars. For the vertical occluder, each of the two bars covered two columns of the game board. For the horizontal occluder, each of the two bars covered three rows of the game board.

In experiment 5, the scene consisted of a night sky with stars and clouds. The size of the night sky image with clouds and stars ($I_{sky\_cloud\_star}$) was 500 × 500 pixels. The night sky images were constructed by a weighted average of multiple images: the color of the background night sky image (*I_sky_*) was changing gradually, from light blue (R = 49, G = 87, and B = 133) to dark blue (R = 10, G = 25, and B = 44) from the upper left to the lower right. The luminance of the original cloud image (*I_cloud_*) was 255 (R = G = B = 255). There were two sizes and luminance of the original star image (*I_star_*). The size of the large stars was 3 × 3 pixels, and the luminance was 171 (R = G = 176, and B = 128). The size of the small stars was 2 × 2 pixels, and the luminance was 213 (R = G = 220, and B = 160). Large and small stars were arranged randomly on the sky.

The occurrence of the large and small stars was balanced in each trial. The night sky image with clouds and stars ($I_{sky\_cloud\_star}$) was then generated by a weighted average of the background sky image (*I_sky_*), the original cloud image (*I_cloud_*), and the original star image (*I_star_*). The weight for the cloud image (*w_cloud_*) was obtained by adjusting a noise map. A Brownian (1/*f*^2^) noise map of the same size as the cloud image was first generated. Then the noise map was shifted downward by subtracting 0.6, after which values smaller than 0 were clamped at 0. The weight for the star image (*w_star_*) was generated by applying Gaussian smoothing with a variance of 0.9 and 1.2 for small and large stars, respectively. In such a way, the night sky with clouds and stars was obtained by:

(1) $$\begin{array}{ccc} & & I_{sky\_cloud\_star}=\left(1-w_{cloud}\right)\\ & & \left(\left(1-w_{star}\right)I_{sky}+\;w_{star}I_{star}\right)+\;w_{cloud}I_{cloud}\; \end{array}$$

### Procedure

In all five experiments, each trial started with a fixation point in the center of the screen without a time limit. After pressing any key, the occluder(s) appeared first, followed by the game board(s) with game pieces after 300 ms. These stimuli disappeared after 2000 ms in experiment 1 and 1250 ms in experiments 2, 3, and 4, followed by different questions to the observers. For the questions in all of the experiments, there was no time limit.

#### Experiment 1: Discrimination of numerosity and confidence judgments

In experiment 1, two game boards with pieces were displayed simultaneously. Observers had to first perform a discrimination task with the question “Which game board has more pieces on it?” They could choose their answer by clicking the “left” or “right” button arranged horizontally. After that, they had to answer the question “How confident are you about your judgement?” by clicking the “High” or “Low” button arranged vertically with the “High” button on top.

For each pair of numerosities, three conditions were investigated: (1) both game boards were fully visible (non-occluded), (2) both game boards were partially occluded (occluded), and (3) one of the game boards was fully visible, and the other was partially occluded (mixed). A total of 11 different numerosities were investigated. The number of visible pieces of the standard stimulus was always 10. For the comparison stimulus, the numbers of visible pieces were two, four, six, eight, nine, 10, 11, 12, 14, 16, and 18. For the mixed condition, the standard was occluded and the comparison was not occluded. Each stimulus was repeated 10 times, with a random arrangement of pieces each time.

In total, there were 352 trials, including 330 test trials (11 numerosities × 3 conditions × 10 repetitions), 11 obscured trials where some of the game pieces were partially obscured in the mixed condition (11 numerosities × 1 condition × 1 repetition), and 11 swapped trials where the standard was not occluded and the comparison was occluded in the mixed condition (11 numerosities × 1 condition × 1 repetition). We added the obscured trials where the game pieces were partially obscured to emphasize for the observers that there might be pieces hidden behind the occluder. We added swapped trials such that the occluded condition did not always contain 10 pieces. Obscured and swapped trials were not analyzed.

At the end of the experiment, observers had to finish a questionnaire regarding the experiment: (1) Did you have the feeling that sometimes the game board was occluded by the green mesh? (2) Did you have the feeling that sometimes the green mesh was occluded by the game board? (3) In your opinion, could some of the pieces have been behind the green mesh?

#### Experiment 2: Numerosity estimation of visible and hidden objects

In experiment 2, observers had to first perform an estimation task: “How many pieces were completely or partially visible on the game board?” by selecting from a drop-down menu (with options ranging from 0 to 40 with a step size of 1). After that, they were asked “Were parts of the game board hidden by the green mesh?” They could answer “Yes” or “No” by clicking on the buttons arranged horizontally. Then, they had to perform a second estimation task: “How many pieces were completely hidden behind the green mesh?” by selecting from the drop-down menu, which had the same setting as the first estimation task.

We inserted the binary judgment task before the estimation of the number of hidden pieces for two reasons. First, we wanted to check whether observers perceived occlusion in all occlusion trials during the experiment. Second, we aimed at leading the observers to consider the occlusion when they estimated the number of hidden pieces subsequently. In terms of internal consistency, responding with yes to the occlusion question should prompt observers to consider the occlusion for the subsequent estimation task.

The number of visible pieces varied from six to 14 with a step size of one, resulting in a total of nine numerosities. Each stimulus was repeated 10 times, with a random arrangement each time. In total, there were 279 trials, including 270 test trials (9 numerosities × 3 conditions × 10 repetitions) and nine obscured trials where the game pieces were partially obscured (9 numerosities × 1 condition × 1 repetition).

The questions in the survey were the same as in experiment 1.

#### Experiment 3: Estimation of occluded area

In experiment 3, observers had to perform one estimation task: “What is the maximum number of pieces that might be behind the occluder?” by dragging a slider ranging from 0 to 117 with a step size of one. One hundred seventeen was the maximum because that was the total number of cells on the game board.

For the occluded relative to the visible area on the game board, seven proportions were investigated: 0.25, 0.36, 0.43, 0.50, 0.58, 0.65, and 0.76. For each proportion, three scales were displayed: 1, 0.75, and 0.5 times the original size. For the occluded condition, each stimulus was repeated 10 times, resulting in 210 trials (7 proportions × 3 scales × 10 repetitions). For the non-occluded condition, each stimulus appeared once, resulting in additional 21 trials (7 proportions × 3 scales × 1 repetition). This resulted in 231 trials in total.

#### Experiment 4: Effect of regularity on numerosity estimation of visible and hidden objects

In experiment 4, observers had to first perform an estimation task: “How many pieces were visible on the game board?” After that, they had to perform a second estimation task: “How many pieces were completely hidden behind the green bars?” Both of the questions could be answered by selecting from a drop-down menu (with options ranging from 0 to 40 with a step size of 1).

The number of visible pieces was five, seven, 10, 12, and 14. For numerosities five, 10, and 12, the shape of the occluder was two vertical bars. For numerosities seven and 14, the shape of the occluder was two horizontal bars. For the irregular condition, we specified five patterns such that the pieces would not form a line. For the regular conditions, the game pieces were arranged in lines either across (regular-across) or outside (regular-outside) the occluder, such that the expected number of hidden pieces was the same for the same numerosities. Each condition was repeated 10 times, resulting in a total of 150 trials (5 numerosities × 3 conditions × 10 repetitions).

At the end of the experiment, observers had to finish a questionnaire with a forced binary choice regarding the experiment: (1) Did you have the feeling that the game board was partially occluded by the green bars? (2) In your opinion, could some of the pieces have been behind the green bars?

#### Experiment 5: Numerosity estimation of visible and hidden objects in a naturalistic scene

In experiment 5, observers had to first perform an estimation task: “How many stars were visible?” by selecting from a drop-down menu (with options ranging from 0 to 40 with a step size of 1). After that, they were asked: “What proportion of the sky was covered by clouds?” by dragging a slider ranging from 0% to 100%. Finally, they had to perform the third estimation task: “How many stars were hidden behind the clouds?” by selecting from the drop-down menu, which had the same setting as the first estimation task.

The number of visible stars varied from six to 14 with a step size of one, resulting in a total of nine numerosities. For each numerosity, the proportion that the sky was covered by the clouds varied from 0.3 to 0.7 with a step size of 0.02, resulting in 21 proportions. Each stimulus occurred only once. In total, there were 189 trials (9 numerosities × 21 proportions).

### Data analysis

Results in this study were analyzed with 1-way repeated-measures ANOVAs and *t*-tests (two tailed). The alpha-level was set to 0.05. A nonlinear least-squares fitting was adopted to fit the models.

In experiment 1, the proportion of choosing the comparison as more numerous was fitted as a function of the difference between the standard and comparison numerosity using a cumulative Gaussian function:

(2) $$\begin{array}{ccc} & & \Psi \left(x;\alpha ;\beta ;\gamma ;\lambda \right)\;\\ & & =\;\gamma +\frac{\left(1-\gamma -\lambda \right)}{2}\left[1+erf\left(\frac{x-\alpha }{\beta \sqrt{2}}\right)\right],\; \end{array}$$

where α indicates the mean of the cumulative Guassian, β indicates the standard deviation, γ is the guess rate, and λ indicates the lapse rate. The parameters that were fitted through the least-square fitting were α, β, and λ, with γ = λ. The point of subjective equality (PSE) was defined as the mean of the underlying cumulative Gaussian.

The proportion of high confidence responses was fitted as a function of the difference between the standard and comparison numerosity using the inverse of the probability density function (PDF) of Gaussian (Gallagher, Suddendorf, & Arnold, 2019; Luna, Serrano-Pedraza, Gegenfurtner, Schütz, & Souto, 2021):

(3) $$\begin{array}{c} C\left(x\right)\;=\;1-ae^{-\frac{1}{2}{\left(\frac{x-\mu }{\sigma }\right)}^{2}}\; \end{array}$$

where µ and σ indicate the mean and standard deviation of Guassian, *a* is the scaling factor of the amplitude and modulates the minimum confidence judgment. The PSE of the confidence judgment was defined as the mean of the underlying Gaussian.

The numerosity estimations in experiments 2, 3, and 4, as well as the estimates of numerosity and hidden proportion in experiment 5, were fitted using a linear function *f*(*x*)  = *p*_1_*x* + *p*_2_, where *p*_1_ indicates the slope and *p*_2_ indicates the intercept.

The precision of the direct estimations in experiments 2 to 5 was measured by the coefficient of variation, that is, the ratio of the standard deviation over the mean.

### Calculation of the ground-truth number of hidden objects

In experiments 1 and 2, the hidden area of the game board (*S_h_*) was obtained by counting the grid cells where pieces would be completely hidden by the occluder. The visible area of the game board (*S_v_*) was obtained by subtracting the completely hidden cells from the total number of cells of the game board.

Assuming a homogeneous distribution (i.e. a constant density), the number of objects hidden behind an occluder (*n_h_* ) can be estimated based on the number of visible objects (*n_v_*) and the area of the visible (*S*_*v* _) and occluded (*S_h_*) parts:

(4) $$\begin{array}{c} n_{h}\;=\;\frac{n_{v}}{S_{v}}S_{h}\; \end{array}$$

We want to point out that $\frac{n_{v}}{S_{v}}$ corresponds to the density of visible objects in the visible area.

### Bayesian model for the estimation of the number of hidden objects

Assuming a homogeneous distribution of objects to the whole surface, the question how many objects are expected to be located in occluded and nonoccluded parts of the surface can be formalized for discrete locations (as in the game board of experiment 2) as the stochastic problem of drawing from an urn with two categories of balls without replacement. The number of visible (*N_v_*) and hidden cells (*N_h_*) corresponds to the number of the two categories of balls in the urn. The total number of objects (*n_v+h_*) corresponds to the number of draws from the urn. The expected number of visible (*n_v_*) and hidden (*n_h_*) objects then corresponds to the resulting number of balls from the two categories. Upper- and lowercase characters denote quantities of the urn and the samples, respectively. A hypergeometric distribution allows to calculate the probability that a certain number of objects (*n_v_*) are visible given the total number objects (*n_v+h_*):

(5) $$\begin{array}{c} P\left(n_{v}|n_{v+h}\right)\;=\;\frac{\left(\begin{array}{c} N_{v}\\ n_{v} \end{array}\right)\cdot \left(\begin{array}{c} N_{v+h}-N_{v}\\ n_{v+h}-n_{v} \end{array}\right)}{\left(\begin{array}{c} N_{v+h}\\ n_{v+h} \end{array}\right)}\; \end{array}$$

However, the total number of objects is unknown to the observers and they are faced with the inverse problem of estimating the total number of objects given the visible number of objects. According to Bayes theorem (for reviews see Ma, Kording, & Goldreich, 2022; Mamassian, Landy, & Maloney, 2002), this posterior distribution (*P*(*n*_*v* + *h*_|*n_v_*)) can be estimated by multiplying the likelihood distribution (*P*(*n_v_*|*n*_*v* + *h*_)) with the prior probability (*P*(*n*_*v* + *h*_)) of the total number of objects, divided by a normalization factor (*P*(*n_v_*)):

(6) $$\begin{array}{c} P\left(n_{v+h}|n_{v}\right)\;=\;\frac{P\left(n_{v}|n_{v+h}\right)\cdot P\left(n_{v+h}\right)}{P\left(n_{v}\right)}\; \end{array}$$

We fitted two different Bayesian models to the data (Figure 3), either with a prior for a constant total number of objects or with a prior for a constant number of hidden objects. These priors were modeled with Gaussian distributions and each model had two free parameters, the mean and the standard deviation of the prior distribution. The Bayesian information criterion (Schwarz, 1978) and relative model weights (Burnham & Anderson, 2002) were calculated for each observer to compare the two models.

## Results

### Experiment 1: Discrimination of numerosity and confidence judgments

In experiment 1 (*N* = 44), two game boards with variable numbers of game pieces appeared simultaneously. For each pair of numerosities, three conditions were investigated: (1) both game boards were fully visible (non-occluded), (2) both game boards were partially occluded (occluded), and (3) one of the game boards was fully visible, and the other was partially occluded (mixed; see Figure 1A). One of the game boards had a fixed number of visible pieces of 10 (standard) and the number of visible pieces on the other game board varied from two to 18 (comparison). In the mixed condition, the standard was occluded and the comparison was not occluded. We calculated the difference between standard and comparison numerosity so that zero indicates that both game boards had the same number of visible pieces. In each trial, observers had to perform two tasks. In the selection task, observers had to choose the game board with more game pieces. In the subsequent confidence task, observers had to report if their confidence about that numerosity selection was high or low.

Figure 1B and C shows psychometric functions of one representative observer. In the selection task, the proportion of choosing the standard as more numerous increased with increasing difference between standard and comparison numerosity (Figure 1B). In the confidence task, the proportion of high confidence responses was high when the absolute difference between standard and comparison was large and minimal when both were similar (Figure 1C).

The amplitude modulation of the confidence function (Equation 3) allows to quantify the overall uncertainty about the numerosity of the game boards (Figure 1E). The confidence amplitude was not significantly different (F(2,84) = 2.38, *p* = 0.099) in the non-occluded (0.71 ± 0.19), occluded (0.73 ± 0.24), and mixed condition (0.76 ± 0.21). This indicates that observers were similarily confident in the mixed and the occluded condition as in the non-occluded condition, although they were objectively missing information due to the occlusion and should have been less confident about their responses than in the non-occluded condition.

In both, the selection and the confidence task, the point of subjective equality (PSE) quantifies which comparison numerosity was perceived equal to the standard numerosity. As expected, the PSE was close to zero in the occluded and the non-occluded conditions because here the standard and the comparison stimuli were actually identical (Figure 1D). In the mixed condition, we expected that observers assumed that the partially occluded standard stimulus had more pieces than the ones that were visible and this should lead to a leftward shift of the psychometric function and a negative PSE. According to Equation 4, the PSE should be at −2.7, indicating that 2.7 pieces are perceived to be hidden behind the occluder. In the selection task, the actual PSE was −1.45 (±1.07), which was significantly smaller than zero (t(43) = −9.00, *p* < 0.001), but also significantly larger than the expected number based on Equation 4 (t(43) = 7.80, *p* < 0.001). This indicates that observers underestimated the number of hidden pieces. Similarly, the point of minimum confidence was −1.99 (±1.08), which was significantly smaller than zero (t(43) = −12.21, *p* < 0.001), but also significantly larger than the expected number (t(43) = 4.33, *p* < 0.001). The PSEs in the selection and the confidence task were highly correlated (r(42) = 0.56, *p* < 0.001). The fact that both, the PSE in the selection task and the point of minimum confidence in the confidence task underestimated the number of hidden pieces is evidence that this underestimation was truly a perceptual effect and not merely a response bias (Gallagher et al., 2019; Luna et al., 2021; Maldonado Moscoso, Cicchini, Arrighi, & Burr, 2020).

This underestimation of the number of hidden objects might have been caused by multiple reasons. First, observers might have not perceived the game board as being occluded at all. However, this is unlikely, because in a post-experiment questionnaire 86.36% of the observers stated that they had the impression that the game board was occluded by the green mesh sometimes, and 95.45% of them stated that some of the pieces could have been hidden behind the green mesh. Second, observers might have underestimated the number of visible pieces (*n_v_*), which would also lead to an underestimation of the number of hidden pieces according to Equation 4. Because the selection task provides only relative numerosity judgments, this cannot be excluded based on the data of experiment 1. Third, observers might have been underestimating the proportion of the occluded relative to the visible area of the game board (*S_h_*/*S_v_*), which would also lead to an underestimation of the number of hidden pieces. Fourth, none of the reasons above holds and observers might not have been estimating the number of hidden pieces according to Equation 4 at all.

We conducted four additional experiments to test these explanations.

### Experiment 2: Numerosity estimation of visible and hidden objects

To investigate whether the underestimation of hidden objects in experiment 1 was caused by an underestimation of the number of visible objects (*n_v_* in Equation 4), we asked observers to report the number of visible and hidden game pieces separately in experiment 2.

**Figure 2.**
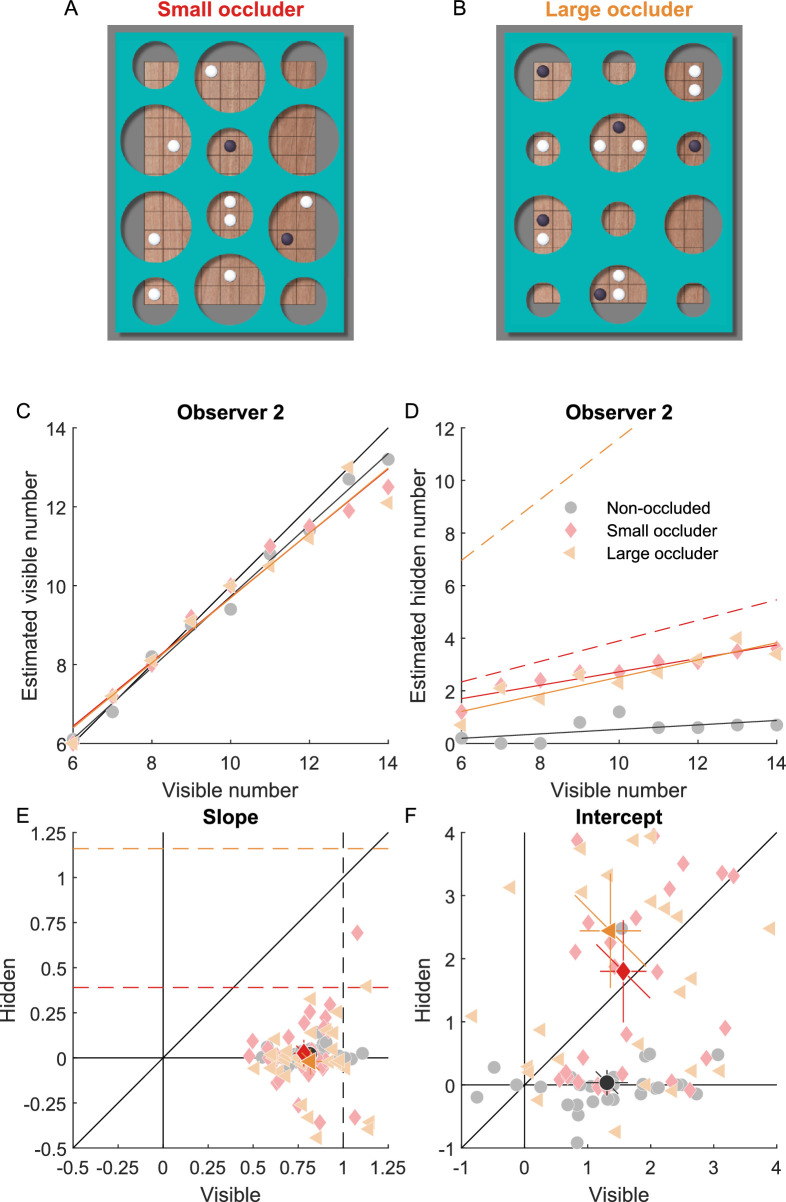
Numerosity estimation of visible and hidden objects. (**A**) Stimulus with a small occluder. (**B**) Stimulus with a large occluder. (**C**) Perceived number of visible pieces as a function of actual number of visible pieces for one representative observer. Colored lines represent linear fits of the data; the black line represents accurate estimation. (**D**) Perceived number of hidden pieces as a function of actual number of visible pieces for one representative observer. Solid lines represent linear fits of the data; dashed lines represent the expected number of hidden pieces obtained from the number of visible pieces and the proportion that the game board was occluded (Equation 4). (**E**) Slope of the linear fit for the number of visible and hidden pieces. Light colors represent individual observers; saturated colors the mean across observers. Error bars represent 95% confidence intervals. The black diagonal represents values with equal effects for visible and hidden pieces. Colored dashed lines represent expected values for the hidden pieces. The black dashed line represents values of unity, where perceptual estimates of the number of visible pieces is accurate. Black solid lines represent values of zero, where perceptual estimates are independent of the number of visible pieces. (**F**) Intercept of the linear fit for the number of visible and hidden pieces. Conventions are the same as in **E**.

**Figure 3.**
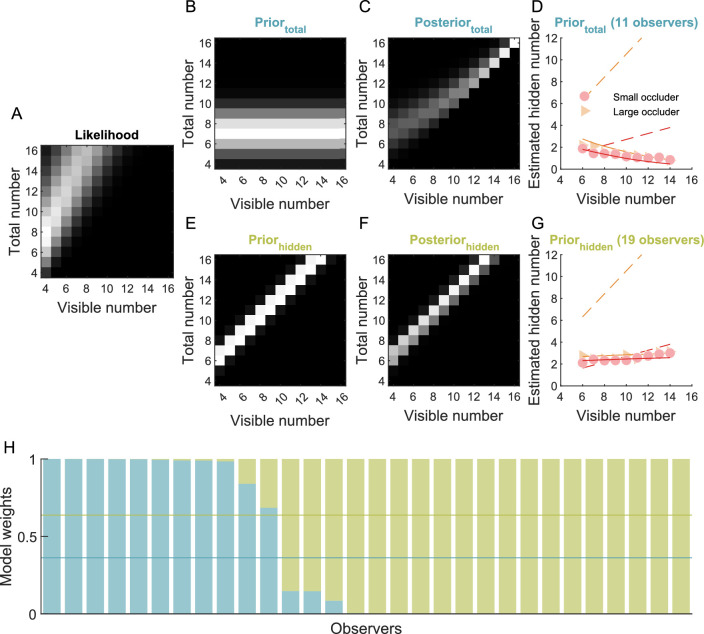
Bayesian model of the estimation of the number of hidden objects. (**A**) Likelihood distribution indicating the probability of a certain number of visible pieces given a total number of pieces (Equation 5). (**B**) Prior distribution with a constant number of total pieces. (**C**) Posterior distribution, combining the likelihood from **A** and the prior from **B** (Equation 6). (**D**) Average perceived number of hidden pieces for those 11 participants whose data are best explained by the model with a constant prior on the total number of pieces. (**E**) Prior distribution with a constant number of hidden pieces. (**F**) Posterior distribution, combining the likelihood from **A** and the prior from **E** (Equation 6). (**G**) Average perceived number of hidden pieces for those 19 participants whose data are best explained by the model with a constant prior on the number of hidden pieces. (**H**) Model weights for all participants. The horizontal lines indicate the average weight for each of the models. **A**, **B**, **C**, **E**, and **F** The grayscale colormap represents probabilities and is normalized for each panel separately to its minimum and maximum to optimize the visibility of individual distributions. In fact, the likelihood distribution is much broader than the prior distribution, such that the posterior is heavily biased towards the prior. **D,** **G** Dashed lines represent the ground-truth value according to Equation 4. Solid lines represent model fits.

In experiment 2 (*N* = 30), only one game board with game pieces appeared in each trial. For each numerosity, three conditions were investigated: (1) the game board was fully visible (non-occluded), (2) the game board was partially occluded by a small occluder as used in experiment 1 (small-occluder; Figure 2A), and (3) the game board was partially occluded by a large occluder with smaller holes (large-occluder; Figure 2B). In each trial, observers had to perform two direct estimation tasks and a binary choice task. In the first direct estimation task, observers had to estimate the number of visible pieces. In the subsequent choice task, observers had to report if they perceived the game board as being occluded or not. Finally, in the second direct estimation task, observers had to estimate the number of hidden pieces.

The binary occlusion task confirmed that the manipulation was successful and observers perceived the occlusion accurately. The average proportion of occlusion reports was 12.96% (±11.86%), 96.11% (±7.98%), and 94.15% (±9.69%) in the conditions of non-occluded, occluded with small and large occluders, respectively.

Figures 2C and D show the numerosity estimation of one representative observer as a function of the actual number of visible pieces. We fitted a linear function to quantify the dependency on the number of visible pieces (slope; Figure 2E) and a general bias due to occlusion (intercept; Figure 2F).

For the estimation of visible pieces, both the slopes and intercepts were not significantly different (slope: F(2,56) = 1.26, *p* = 0.291; intercept: F(2,56) = 0.44, *p* = 0.649) between the non-occluded (slope 0.81 ± 0.13, intercept 1.31 ± 0.89), small-occluder (slope 0.78 ± 0.15, intercept 1.57 ± 0.98), and large-occluder (slope 0.82 ± 0.17, intercept 1.36 ± 1.30) conditions. Slopes around 0.8 and intercepts around one in all three conditions indicate that observers slightly overestimated the number of visible pieces for small numerosities and slightly underestimated for large numerosities. Nevertheless, in general, observers were able to estimate the number of visible pieces quite accurately, making it clear that the underestimation of hidden objects in experiment 1 was not caused by the underestimation of visible objects.

For the estimation of hidden pieces, we would expect that for the same size of the occluder, observers assume more pieces were hidden behind the occluder with larger numerosities, and for the same numerosity, more pieces were hidden behind the large than the small occluder. According to Equation 4, the expected slope of the linear fit should be 0.27 and 1.05 for the small and the large occluder, respectively. Although the slopes were significantly different (t(29) = −2.30, *p* = 0.029) in the small-occluder (0.03 ± 0.20) and large-occluder (−0.02 ± 0.19) conditions, both were significantly smaller than expected (small-occluder: t(29) = −6.74, *p* < 0.001; large occluder: t(29) = −30.39, *p* < 0.001). The intercepts were significantly different from each other (t(29) = 2.58, *p* = 0.015) and significantly larger than 0 in the small-occluder (1.80 ± 2.17, t(29) = 4.54, *p* < 0.001) and large-occluder (2.44 ± 2.43, t(29) = 5.51, *p* < 0.001) conditions. These intercepts were roughly consistent with the PSE shifts in experiment 1, of about −1.45 and −1.99 in the selection and the confidence task, respectively. In the non-occluded condition, slopes (0.02 ± 0.04, t(29) = 2.69, *p* = 0.011) and intercepts (0.04 ± 0.54, t(29) = 0.36, *p* = 0.723) were close to zero, indicating that observers did not hallucinate hidden pieces when there was no occlusion.

Interestingly, some observers showed positive and some observers showed negative correlations between the estimation of the number of hidden pieces and the number of visible pieces (Figure 2E). We evaluated if the overall strong underestimation of the number of hidden pieces and the different estimation patterns would be compatible with a Bayesian model combining information about the number of visible pieces and the occluder with a prior (see Figure 3). We compared two models with either a constant prior on the total number of pieces or a constant prior on the number of hidden pieces. The model with a constant prior on the total number of pieces predicts a decrease in the estimation of hidden pieces with increasing number of visible pieces and was the more likely model for 11 of 30 observers (average model weight 36%). For these observers, the average prior (7.21 ± 4.07) slightly underestimated the average number of ten visible pieces. The model with a constant prior on the number of hidden pieces predicts an increase in the estimation of hidden pieces with increasing number of visible pieces and was the more likely model for 19 of 30 observers (average weight 64%). For these observers, the prior was 2.48 ± 1.45 hidden pieces. Hence, the data of most observers was compatible with a constant prior of about two hidden pieces.

Overall, the results of experiment 2 indicate that observers assumed a constant number of about two hidden pieces, regardless of the size of the occluder and the number of visible pieces. According to Equation 4, the estimation of hidden pieces depends on the estimation of visible pieces (*n_v_*) and the proportion of the occluded relative to the visible area of the game board (*S_h_*/*S_v_*). Experiment 2 showed that observers were able to accurately estimate the number of visible pieces *n_v_* but still were very inaccurate in the estimation of the number of hidden pieces. Possibly, they were unable to estimate the relative proportion of occluded and visible area of the game board (*S_h_*/*S_v_*) (Palmer, Brooks, & Lai, 2007; Scherzer & Ekroll, 2015). To investigate whether the underestimation was caused by underestimating the occlusion, we asked observers to report the proportion of the occluded area in experiment 3.

### Experiment 3: Estimation of occluded area

**Figure 4.**
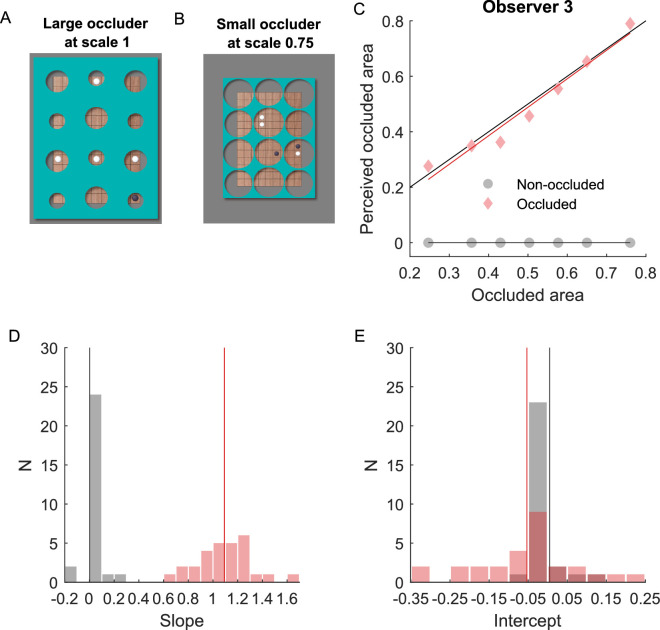
Estimation of occluded area. (**A**) Stimuli of the original size as in experiments 1 and 2. (**B**) Stimuli scaled 0.75 times relative to the original size. (**C**) Estimation of the proportion of the occluded area as a function of the actual occluded area for one representative observer. Solid lines represent linear fits of the data; dashed lines represent values with an accurate match between estimated and actual proportion of the occluded area. (**D**) Histogram of the slope of the linear fit for the maximum number of hidden pieces. Thin vertical lines indicate the mean across observers. (**E**) Histogram of the intercept of the linear fit for the maximum number of hidden pieces. Thin vertical lines indicate the mean across observers.

In experiment 3 (*N* = 28), the proportion of occluded area and the spatial scale of the game board was varied (Figures 4A, B).

For each proportion of occluded area, two conditions were investigated: (1) the game board was fully visible (non-occluded), and (2) the game board was partially occluded (occluded). In each trial, observers had to perform an estimation task, where they had to estimate the maximum number of pieces that might be behind the occluder. This estimation was converted into a proportion of occluded area to compare it directly with the actual proportion of occluded area.

Figure 4C shows the data of one representative observer. The estimated proportion of the occluded area was analyzed as a linear function of the actual occluded area. In the occluded condition, the slope (Figure 4D) of the linear fit was slightly larger than one (1.09 ± 0.22, t(27) = 2.24, *p* = 0.034), and the intercept (Figure 4E) was slightly smaller than 0 (−0.05 ± 0.13, t(27) = −2.22, *p* = 0.035). This indicates that observers were able to accurately estimate the occluded area. Hence, the underestimation of hidden pieces in experiments 1 and 2 was not caused by underestimating the proportion of the occluded relative to the visible area of the game board (*S_h_*/*S_v_*). A remaining explanation for the inaccurate estimation of the number of hidden pieces would be that observers simply did not pay attention to the visible pieces for their estimation of hidden pieces. Therefore, we tested in experiment 4, if the arrangement of the visible pieces modulates the estimation of hidden pieces.

### Experiment 4: Effect of regularity on numerosity estimation of visible and hidden objects

Because previous studies showed that simple lines and contours are perceptually completed behind occluders (Davis & Driver, 1994; Kanizsa, 1979; Michotte, Thinès, & Crabbé, 1964), the irregular arrangement of the game pieces might have been too complex for accurate perceptual completion to occur. To study if the estimation of the number of hidden pieces is modulated by the regularity of the visible pieces’ arrangement, we compared different regular and irregular arrangements in experiment 4.

**Figure 5.**
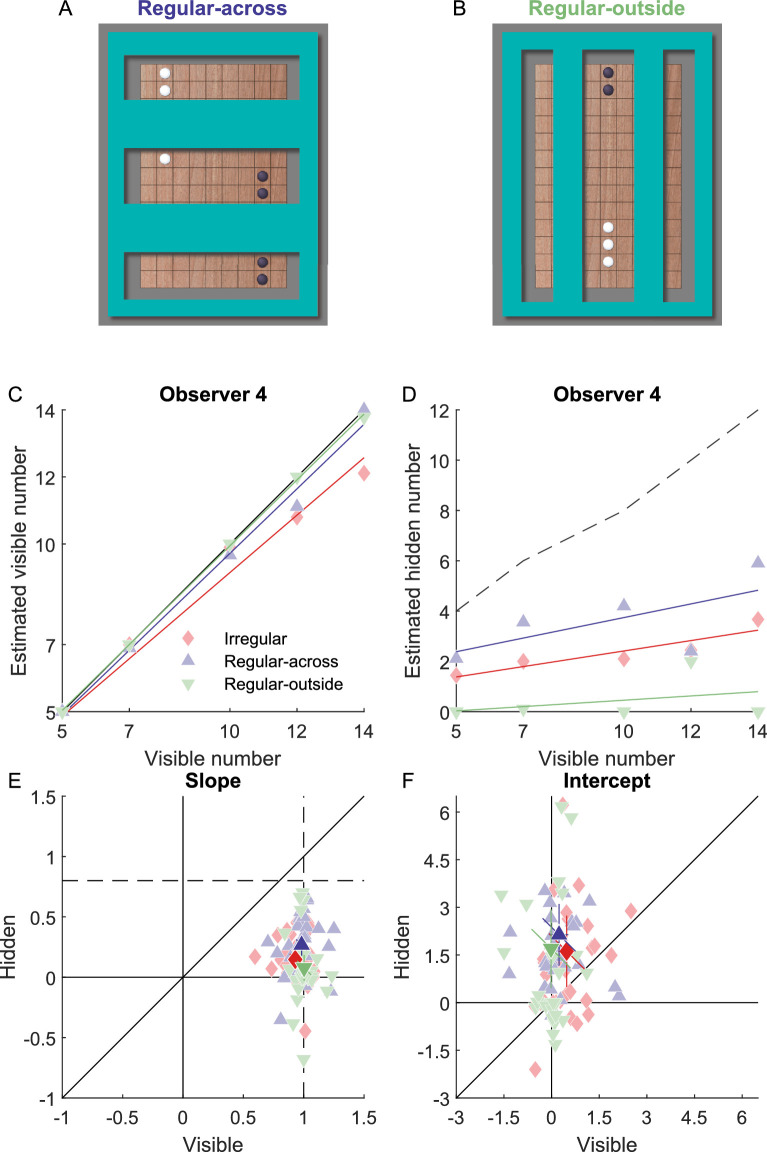
Effect of regularity on numerosity estimation of visible and hidden objects. (**A,** **B**) Stimuli in the regular-across (left) and regular-outside (right) conditions. (**C**) Perceived number of visible pieces as a function of actual number of visible pieces for one representative observer. Colored lines represent linear fits of the data; the black line represents accurate estimation. (**D**) Perceived number of hidden pieces as a function of actual number of visible pieces for one representative observer. Solid lines represent linear fits of the data; dashed lines represent the expected number of hidden pieces obtained from the number of visible pieces and the proportion that the game board was occluded. (**E**) Slope of the linear fit for the number of visible and hidden pieces. (**F**) Intercept of the linear fit for the number of visible and hidden pieces. **E,** **F** Conventions are the same as in Figure 2E.

In experiment 4 (*N* = 29), one game board with game pieces appeared. For each numerosity, three conditions were investigated: (1) pieces arranged irregularly, like in experiments 1 and 2 (irregular), (2) pieces arranged regularly, forming a line across the occluded area (regular-across; Figure 5A), (3) pieces arranged regularly, forming a line outside of the occluded area (regular-outside; Figure 5B). To facilitate the generation of regular patterns across and outside the occluder, the shape of the occluder was changed to vertical or horizontal bars. In the irregular and regular-outside conditions, the expected number of hidden pieces was obtained according to Equation 1. In the regular-across condition, the expected hidden pieces are the ones along the straight path formed by the visible pieces. We arranged the visible pieces such that the expected number of hidden pieces was the same in both regular conditions across and outside the occluder. In each trial, observers had to perform two direct estimation tasks like in experiment 2 to estimate the number of visible and hidden pieces separately. Like in experiment 2, we analyzed the estimated number of visible and hidden pieces as a linear function of the actual number of visible pieces (Figures 5C, D).

For the estimation of visible pieces, the slopes were not significantly different in the three conditions (F(2,56) = 2.60, *p* = 0.084). They were slightly smaller than 1 in the irregular condition (0.93 ± 0.11, t(29) = −3.43, *p* = 0.002), but not in the regular-across (0.98 ± 0.12, t(29) = −0.80, *p* = 0.428) and regular-outside (1.00 ± 0.08, t(29) = 0.24, *p* = 0.816) conditions. The intercepts were significantly different in the three conditions (F(2,56) = 3.29, *p* = 0.044). They were slightly larger than zero in the irregular condition (0.48 ± 0.71, t(29) = 3.67, *p* < 0.001), but not in the regular-across (0.23 ± 0.78, t(29) = 1.64, *p* = 0.111) and regular-outside (−0.02 ± 0.57, t(29) = −0.17, *p* = 0.867) conditions. This indicates that the estimation of the number of visible pieces was accurate, for both irregular and regular patterns of pieces.

However, the regularity affected the estimation of the number of hidden pieces. For the estimation of hidden pieces, the slope is expected to be 0.8 according to Equation 4. In the estimation task, the slopes were significantly different from each other (F(2,56) = 3.48, *p* = 0.038) and significantly smaller than 0.8 in the irregular (0.15 ± 0.20, t(29) = −17.88, *p* < 0.001), regular-across (0.27 ± 0.24, t(29) = −12.36, *p* < 0.001), and regular-outside (0.08 ± 0.29 , t(29) = −13.83, *p* < 0.001) conditions. The intercepts in the irregular, regular-across and regular-outside conditions were not significantly different from each other (F(2,56) = 0.58, *p* = 0.565). All intercepts were significantly larger than zero (irregular: 1.62 ± 3.00, t(29) = 2.95, *p* = 0.006; regular-outside: 1.70 ± 3.32, t(29) = 2.81, *p* = 0.009; and regular-across: 2.14 ± 2.60, t(29) = 4.51, *p* < 0.001).

The slope in the regular-across condition was smaller than expected but higher than that in the other two conditions. This indicates that when the visible pieces formed a line across the occluded area of the game board, the underestimation of hidden pieces was still present, but reduced. The slope in the regular-outside condition was even smaller than that in the irregular condition, indicating that when the visible pieces formed a line separately from the occluded areas, the underestimation was even more pronounced compared to irregular patterns. This modulation by the arrangement of the visible pieces clearly shows that observers used the number and arrangement of the visible pieces and were not simply ignoring them when estimating the number of hidden pieces.

Nevertheless, observers did not use the information about the number of visible pieces and the area of occlusion to accurately estimate the number of hidden pieces in experiments 1, 2, and 4. One remaining explanation might be that observers did not share the assumption that the density of pieces is constant across the artificial, man-made game board. If the pieces are not uniformly distributed across the game board, the number of hidden pieces cannot be sensibly estimated using Equation 4. To this end, we tested a more naturalistic scene in the final experiment 5.

### Experiment 5: Numerosity estimation of visible and hidden objects in a naturalistic scene

**Figure 6.**
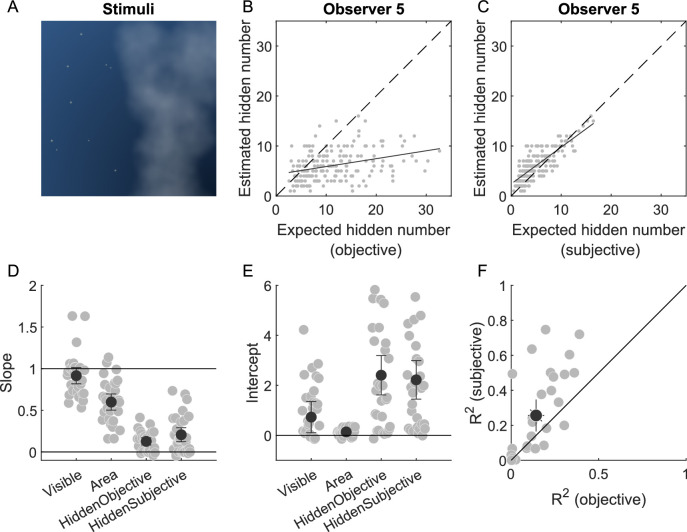
Numerosity estimation of visible and hidden objects in a naturalistic scene. (**A**) Stimuli of a night sky with stars and clouds. Observers had to report the number of visible stars, the proportion of the sky covered by clouds and the number of stars hidden by clouds. (**B**) Perceived number of hidden stars as a function of the objectively expected number of hidden stars for one representative observer. The objectively expected number of hidden stars was obtained from the actual number of visible stars and the actual proportion of the sky covered (Equation 4). The solid line represents the linear fit of the data. (**C**) Perceived number of hidden stars as a function of subjectively expected number of hidden stars for one representative observer. The subjectively expected number of hidden stars was obtained from the perceived number of visible stars and the perceived proportion of occlusion (Equation 4). The solid line represents the linear fit of the data. (**D**) Slope of the linear fit for the perceived number of visible stars, the perceived proportion of occlusion, the perceived number of hidden stars as a function of objectively or subjectively expected number of hidden stars. Light colors represent individual observers; saturated colors the mean across observers. Error bars represent 95% confidence intervals. The black solid line represents values of unity, where perceptual estimates are accurate. (**E**) Intercept of the linear fit for the same quantities as in **D**. Conventions are the same as in **D**. (**F**) Coefficient of determination (R²) of the linear fit for the perceived number of hidden stars (objective and subjective). Error bars represent 95% confidence intervals. The black diagonal represents values with equal effects for objective and subjective expectations.

To investigate whether the underestimation of hidden pieces in the previous experiments was caused by assuming a non-uniform distribution in an artificial, man-made scene, we presented a more naturalistic scene, consisting of a night sky with randomly arranged stars and clouds covering parts of the sky (see Figure 6A). In experiment 5 (*N* = 28), an image of the night sky with stars and clouds appeared in each trial, and observers had to perform three direct estimation tasks: First, observers had to estimate the number of visible stars. Second, observers had to report the proportion of the sky that was covered by the clouds. Third, observers had to estimate the number of hidden stars.

The perceived number of visible stars and the proportion of occlusion were fitted with a linear function (see Figure 6D, E) like in experiments 2 and 3. For the estimation of visible stars, the slope was not significantly different from 1 (0.92 ± 0.27, t(27) = −1.640, *p* = 0.113) and the intercept was not significantly different from 0 (0.73 ± 1.73, t(27) = 2.224, *p* = 0.305). This indicates that observers were able to estimate the number of visible stars accurately. For the estimation of the proportion of occlusion, the slope was 0.61 (±0.27) and significantly smaller than 1 (t(27) = −7.853, *p* < 0.001). The intercept was around 0.13 (±0.13) and significantly larger than 0 (t(27) = 5.190, *p* < 0.001). This indicates that observers underestimated the proportion of the sky covered by clouds.

For the numerosity estimation of hidden stars, we would expect that observers estimate the number of hidden stars based on the number of visible stars and the proportion of the sky covered by clouds (Equation 4). We fitted a linear function to quantify the relationship between the estimates and the objectively expected number of hidden stars. Accurate estimates would be represented by a slope of one and an intercept of zero. The slope was 0.13 (±0.12) and significantly smaller than 1 (t(27) = −39.954, *p* < 0.001). The intercept was 2.55 (±2.12) and significantly larger than 0 (t(27) = 6.347, *p* < 0.001). This indicates that observers underestimated the number of hidden stars relative to the objectively expected number according to Equation 4.

Given that the observers underestimated the occluded area in the second estimation task, it might be that the underestimation of the number of hidden stars was merely a consequence of the underestimation of occlusion. To test this hypothesis, we further analyzed the estimated number of hidden stars as a linear function of the subjectively expected number of hidden stars, using the trial-by-trial estimations of the number of visible stars and the proportion of occlusion. This significantly increased (t(27) = −3.44, *p* = 0.002) the explained variance from 0.15 (±0.13) with the objective prediction to 0.27 (±0.25) with the subjective prediction (see Figure 6F). However, the slope was with 0.21 (±0.23) still significantly smaller than 1 (t(27) = −18.572, *p* < 0.001), and the intercept was with 2.37 (±2.06) still significantly larger than 0 (t(27) = 6.080, *p* < 0.001). This indicates that observers still heavily underestimated the number of hidden stars even when taking into account their individual misestimations of the number of visible stars and the proportion of occlusion (Appendix).

## Discussion

In five experiments, we showed that human observers do not accurately estimate the number of hidden objects. The results from a numerosity discrimination task in experiment 1 showed that observers underestimated the number of hidden objects but were overconfident in the presence of occlusion. The underestimation was not caused by observers having overlooked the occlusion, because trial-by-trial questions and post-experiment questionnaires showed that most of them were aware of the occlusion. The results from the direct estimation tasks in experiments 2 and 3 showed that observers were able to accurately estimate the number of visible objects and the proportion of occlusion. Hence, all the necessary information to accurately estimate the number of hidden objects was available to them, but that did not result in accurate estimates according to Equation 4. Experiment 4 showed that observers were not simply ignoring the visible objects because their arrangement modulated the estimation of hidden objects. Regular arrangements across occlusion led to larger and regular arrangements outside of the occlusion led to smaller numbers of hidden objects compared to irregular arrangements. Experiment 5 showed that even in a more naturalistic scene with uniformly distributed objects, observers still underestimated the number of hidden objects.

A remaining question of course is why observers’ estimations were inaccurate. On a behavioral level, one could argue that humans simply do not assume a constant density of objects in the world. Without that assumption, it would be nonsensical to try to estimate the number of hidden objects based on the number of visible objects. However, it is not obvious why our visual system should not subscribe to this assumption. In most cases, the visible part of the environment should be the best guess for its non-visible parts. In addition, at a lower level, for simple contours and surfaces, the visual system follows the assumption that nothing special happens behind occluders and that information from the surround can be filled-in (de Weerd, 2006; Komatsu, 2006). Why does it not follow the same assumption and rules for more complex and irregular arrangements of objects? First, it simply might be an issue of complexity, so that information is not completed at all processing levels, but only for simple low-level features, such as orientation or color. Second, it might be an issue of receptive field size. (Neural) Models of perceptual completion typically assume that neurons with receptive fields at the gap receive lateral input from neurons with receptive fields outside of the gap (Komatsu, 2006) to complete the missing information in the gap. It is known that receptive field size increases along the visual hierarchy (Smith, Singh, Williams, & Greenlee, 2001). Completion might fail, if receptive fields are larger than the gaps in information. With respect to these first two points, there is contradictory evidence about the question whether numerosity is a low- or a high-level feature. Some studies found numerosity representations primarily at late processing stages in prefrontal and parietal cortex (Nieder, Freedman, & Miller, 2002; Roitman, Brannon, & Platt, 2007), whereas others found numerosity-related activity already in early visual cortex (DeWind, Park, Woldorff, & Brannon, 2019; Fornaciai, Brannon, Woldorff, & Park, 2017). Third, although we showed that observers have all the necessary quantities to estimate the number of hidden objects, combining them like in Equation 4 might be difficult. In particular, calculating the density of the visible objects might not be something that is done intuitively. Because numerosity and density are necessarily intertwined visual features, there has been an intensive debate about whether numerosity is a primary visual attribute and under which circumstances humans use numerosity or texture density as quantitative visual cues (Burr & Ross, 2008; Dakin, Tibber, Greenwood, Kingdom, & Morgan, 2011; Durgin, 2008). Our results clearly show that observers did not use density to solve the task, because using the density of objects in the visible parts of the stimulus would have automatically resulted in accurate choices in experiment 1. Furthermore, the estimation of hidden objects was independent of the size of the occluder and therefore also independent of the density of visible objects, which covary. Hence, our results are consistent with other findings that numerosity can be considered a primary visual attribute (Cicchini, Anobile, & Burr, 2016) and that it takes precedence over density in our paradigm even when it is not useful for the task. Fourth and finally, it might be an issue of uncertainty. The Bayesian model indicates that the uncertainty about the number of hidden objects might be so large that estimates are completely dominated by the prior (either about the number of all or of hidden objects).

Smaller inaccuracies in the estimation of numerosity of visible objects have been reported in various paradigms: numerosity is underestimated in front-planes compared to back-planes at low densities (Schütz, 2012), in back-planes compared to front-planes at high densities (Scaccia & Langer, 2019), in the periphery compared to the fovea (Balas, 2016; Valsecchi, Toscani, & Gegenfurtner, 2013; but see Hübner & Schütz, 2017) in high-contrast elements compared to low-contrast elements (Lei & Reeves, 2018) and during saccadic eye movements (Binda, Morrone, Ross, & Burr, 2011). The underestimation of numerosity in back-planes at high densities was interpreted as an effect of occlusion from the front-plane (Scaccia & Langer, 2019).

Our results relate to a perceptual illusion, called “illusion of empty space,” where a partially occluded scene is perceived to be empty behind the occluder (Ekroll, 2019; Ekroll, Sayim, van der Hallen, & Wagemans, 2016; Luna et al., 2021; Michotte, Thinès, & Crabbé, 1964; Øhrn et al., 2019). Previous studies showed that the illusion of absence is not just due to the lack of retinal stimulation, but rather a result of perceptual completion (Ekroll, 2019). The illusion can lead for instance to perceive an object to magically float in space, because the possibility that the object is actually resting on another, completely hidden object is neglected. However, our results go beyond the illusion of absence, because in our case there is sensory information about the presence of objects, which, however, does not seem to be used to accurately estimate the number of hidden objects. This is also reminiscent of the development of object permanence, where very young infants fail to represent the presence of objects that disappear from their view behind occluders (Munakata, McClelland, Johnson, & Siegler, 1997). In our case, adults seem to fail to represent the presence of irregularly arranged objects behind occluders.

Last but not least, our findings are also relevant for the field of metacognition (Mamassian, 2016). The results in the confidence task in experiment 1 showed that observers were similar confident to discriminate numerosity when comparing two fully visible scenes, two partially occluded or one partially occluded and one visible scene. Given that they objectively lacked information for partially occluded scenes, this can be considered as an error of metacognitive confidence, where observers were overconfident in their inferences about the occluded area. This might be linked to two other phenomena of metacognitive distortions. The first one is about natural scotomata where no sensory information is processed, such as the blind-spot and the foveal rod scotoma. Both gaps are perceptually completed (Ehinger, Häusser, Ossandón, & König, 2017; Gloriani & Schütz, 2019; Ramachandran, 1992) and humans are overconfident for the inferred information compared to sensory information at other locations (Ehinger et al., 2017; Gloriani & Schütz, 2019). The second one is about the limitations of peripheral vision, which is characterized by lower resolution and larger spatial distortions and uncertainty than foveal vision (for reviews see Rosenholtz, 2016; Stewart, Valsecchi, & Schütz, 2020; Strasburger, Rentschler, & Jüttner, 2011). Strikingly, the fidelity of peripheral vision is overestimated and that misestimation has been interpreted as subjective inflation (Knotts, Odegaard, Lau, & Rosenthal, 2019; Odegaard, Chang, Lau, & Cheung, 2018; Solovey, Graney, & Lau, 2015), where observers believe to perceive more than they actually can see rather than perceptual filling-in, where missing information is (accurately) completed. In this light, our results could be interpreted as a phenomenon of inflation rather than perceptual completion because our observers were confident about their inaccurate estimations of the number of hidden objects. This is consistent with the recent finding that metacognitive confidence relates to the consistency rather than the accuracy of responses (Caziot & Mamassian, 2021).

## Figures and Tables

**Figure A1.**
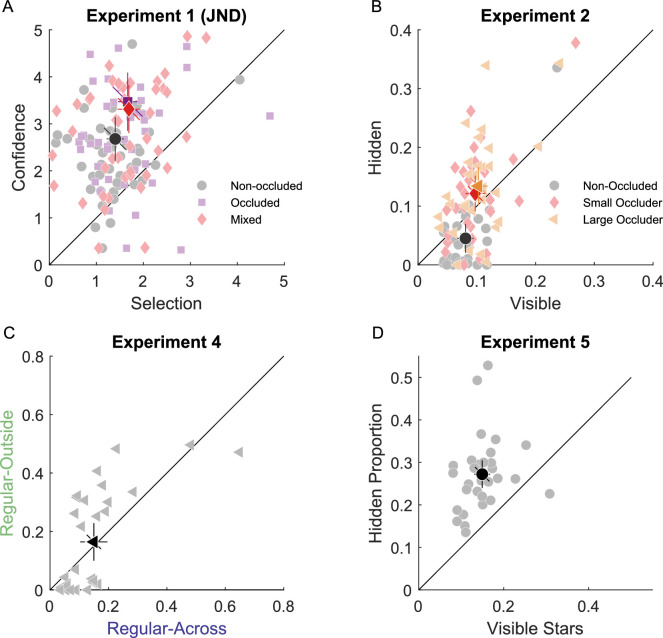
Precision of responses. **A**) JNDs in the selection and confidence task of Experiment 2. Light colors represent individual observers; saturated colors the mean across observers. Error bars represent 95% confidence intervals. **B)** Coefficient of variation of the estimation of the number of visible and hidden pieces in Experiment 2. Light colors represent individual observers; saturated colors the mean across observers. Error bars represent 95% confidence intervals. **C**) Coefficient of variation in the estimation of hidden pieces in the regular-outside and the regular-across conditions of Experiment 4. Light gray represent individual observers; black the mean across observers. Error bars represent 95% confidence intervals. **D**) Coefficient of variation in the estimation of the number of visible stars and the proportion that the sky was covered. Conventions are the same as in C.

## Appendix

### Underestimation of the number of hidden pieces as a function of perceived number of visible pieces in experiments 1 and 2

Because the observers had no access to the actual number of visible pieces, they could only use the perceived number of visible pieces to estimate the number of hidden pieces. An underestimation of the number of visible pieces should therefore also lead to an underestimation of the number of hidden pieces. Here, we analyzed if the underestimation of visible pieces could fully account for the underestimation of hidden pieces.

Given the estimation of visible number in experiment 2, the perceived number of the visible standard in experiment 1 would be nine (= 0.8 × 10 + 1), leading to an expected PSE of −2.4 for the mixed-condition according to Equation 1. This was quite close to the expected PSE of −2.7, obtained with the actual number of visible pieces. Indeed, the empirical PSE in experiment 1 was still significantly smaller than −2.4 (t(43) = 5.93, *p* < 0.001).

In experiment 2, the average perceived number of visible pieces was 5.8, 6.6, 7.4, 8.2, 9.0, 9.8, 10.6, 11.4, and 12.2. Using these values to calculate the expected number of hidden pieces according to Equation 1, the expected slope would be 0.22 and 0.84 for the small and large occluder, respectively. Again, these values were quite close to the expected slope of 0.27 and 1.05, obtained with the actual number of visible pieces. The empirical data in experiment 2 was significantly smaller than 0.22 in the small-occluder (t(29) = −5.36, *p* < 0.001) and smaller than 0.84 in the large occluder condition (t(29) = −24.43, *p* < 0.001).

This additional analysis clearly shows that the small underestimation of the number of visible pieces cannot account for the larger underestimation of the number of hidden pieces.

### Precision of responses

In experiment 1, the just-noticeable difference (JND) as the standard deviation of the Gaussian functions (Equations 2 and 3) can be taken as estimate for the precision in the perceptual discrimination and the confidence judgments. In the selection task, the JND was not significantly different (F(2,84) = 1.64, *p* = 0.201) in the non-occluded (1.44 ± 0.62), occluded (1.60 ± 0.55) and mixed condition (1.71 ± 0.88). In the confidence task, the JND was significantly different (F(2,84) = 3.56, *p* = 0.033) in the non-occluded (2.78 ± 1.66), occluded (3.62 ± 2.17), and mixed condition (3.43 ± 1.80). Hence, the precision was not affected by occlusion in the perceptual discrimination task, but in the confidence task. This indicates that observers were less certain about their confidence choices under conditions of occlusion.

In the direct estimation tasks of experiments 2 to 5, we analyzed the coefficient of variation (CV) as measure of precision. The CV normalizes the standard deviation of the responses by their mean. It is a matter of debate if the CV and the Weber fraction are constant for different stimulus numerosities and therefore obey Weber's law (Anobile, Cicchini, & Burr, 2014; Testolin & McClelland, 2021). However, for the small stimulus numerosities used in our experiment, the CV is typically rather constant between 0.1 and 0.3 (Anobile et al., 2014; Anobile, Castaldi, Moscoso, Burr, & Arrighi, 2020; Pomè, Anobile, Cicchini, & Burr, 2019; Testolin & McClelland, 2021).

In experiment 2 (Figure A1A), the CVs of the number estimation of the visible pieces were not different from each other (F(2,56) = 2.79, *p* = 0.070), but in the typical range of Weber fractions of about 0.1 (Anobile et al., 2020; Pomè et al., 2019; non-occluded: 0.09 ± 0.04; smaller occluder: 0.10 ± 0.04; large-occluder: 0.10 ± 0.04). The CVs of the number estimation of hidden pieces (F(2,56) = 19.30, *p* < 0.001) were significantly different from each other, but also close to 0.1 in the non-occluded (0.05 ± 0.07), small-occluder (0.12 ± 0.08), and large-occluder (0.13 ± 0.09) conditions.

In experiment 3 (Figure A1B), the CV in the occluded condition was at the upper end of the typical range (0.25 ± 0.07), whereas the CV in the non-occluded condition was close to zero (0.02 ± 0.04).

In experiment 4, CVs in the estimation of hidden pieces were not significantly different (F(2,56) = 0.86, *p* = 0.429) and again in the typical range in the irregular (0.16 ± 0.12), regular-across (0.15 ± 0.13), and regular-outside (0.17 ± 0.18) conditions (Figure A1C).

It can be seen that although observers in experiments 2, 3, and 4 were inaccurate in the estimation of the number of hidden pieces, they were rather consistent in their estimations.

In experiment 5, CVs in the estimation of the number of visible stars and the proportion of occlusion were significantly different (t(27) = −6.94, *p* < 0.001). The estimation of the number of visible stars was in the typical range (0.15 ± 0.05). The estimation of the hidden proportion was higher (0.27 ± 0.09) but very similar to the CV of the same task in experiment 3. This shows that the observers were consistent in their estimations of the number of visible stars and the proportion of occlusion in the naturalistic scene.

## References

[bib1] Anobile, G., Castaldi, E., Moscoso, P. A. M., Burr, D. C., & Arrighi, R. (2020). “Groupitizing”: a strategy for numerosity estimation. *Scientific Reports,* 10(1), 13436. 32778672 10.1038/s41598-020-68111-1 PMC7417557

[bib2] Anobile, G., Cicchini, G. M., & Burr, D. C. (2014). Separate mechanisms for perception of numerosity and density. *Psychological Science,* 25(1), 265–270. 24270462 10.1177/0956797613501520

[bib3] Balas, B. (2016). Seeing number using texture: How summary statistics account for reductions in perceived numerosity in the visual periphery. *Attention, Perception & Psychophysics,* 78(8), 2313–2319. 10.3758/s13414-016-1204-6 PMC6070299 27613743

[bib4] Binda, P., Morrone, M. C., Ross, J., & Burr, D. C. (2011). Underestimation of perceived number at the time of saccades. *Vision Research,* 51(1), 34–42. 20934445 10.1016/j.visres.2010.09.028

[bib5] Burnham, K. P., & Anderson, D. R. (Ed.). (2002). *Model selection and multimodel inference: A practical information-theoretic approach* (2nd ed.). New York, NY: Springer.

[bib6] Burr, D., & Ross, J. (2008). A visual sense of number. *Current Biology: CB,* 18(6), 425–428. 18342507 10.1016/j.cub.2008.02.052

[bib7] Caziot, B., & Mamassian, P. (2021). Perceptual confidence judgments reflect self-consistency. *Journal of Vision,* 21(12), 8. 10.1167/jov.21.12.8 PMC8606852 34792536

[bib8] Cicchini, G. M., Anobile, G., & Burr, D. C. (2016). Spontaneous perception of numerosity in humans. *Nature Communications,* 7, 12536. 10.1038/ncomms12536 PMC4999503 27555562

[bib9] Dakin, S. C., Tibber, M. S., Greenwood, J. A., Kingdom, F. A. A., & Morgan, M. J. (2011). A common visual metric for approximate number and density. *Proceedings of the National Academy of Sciences of the United States of America,* 108(49), 19552–19557. 22106276 10.1073/pnas.1113195108 PMC3241748

[bib10] Dakin, S. C., & Watt, R. J. (1997). The computation of orientation statistics from visual texture. *Vision Research,* 37(22), 3181–3192. 9463699 10.1016/s0042-6989(97)00133-8

[bib11] Davis, G., & Driver, J. (1994). Parallel detection of Kanizsa subjective figures in the human visual system. *Nature,* 371(6500), 791–793. 7935838 10.1038/371791a0

[bib12] de Leeuw, J. R. (2015). jsPsych: a JavaScript library for creating behavioral experiments in a Web browser. *Behavior Research Methods,* 47(1), 1–12. 24683129 10.3758/s13428-014-0458-y

[bib13] de Weerd, P. (2006). Perceptual filling-in: more than the eye can see. In *Progress in Brain Research. Visual Perception - Fundamentals of Vision: Low and Mid-Level Processes in Perception* (pp. 227–245). New York, NY: Elsevier. 10.1016/S0079-6123(06)54012-9 17010714

[bib14] DeWind, N. K., Park, J., Woldorff, M. G., & Brannon, E. M. (2019). Numerical encoding in early visual cortex. *Cortex; A Journal Devoted to the Study of the Nervous System and Behavior,* 114, 76–89. 29983159 10.1016/j.cortex.2018.03.027 PMC6170729

[bib15] Durgin, F. H. (2008). Texture density adaptation and visual number revisited. *Current Biology: CB,* 18(18), R855–R856; author reply R857-8. 18812077 10.1016/j.cub.2008.07.053

[bib16] Ehinger, B. V., Häusser, K., Ossandón, J. P., & König, P. (2017). Humans treat unreliable filled-in percepts as more real than veridical ones. *eLife,* 6, e21761. 28506359 10.7554/eLife.21761 PMC5433845

[bib17] Ekroll, V. (2019). Illusions of Imagery and Magical Experiences. *i-Perception,* 10(4), 2041669519865284. 31565210 10.1177/2041669519865284 PMC6755141

[bib18] Ekroll, V., Sayim, B., van der Hallen, R., & Wagemans, J. (2016). Illusory Visual Completion of an Object's Invisible Backside Can Make Your Finger Feel Shorter. *Current Biology: CB,* 26(8), 1029–1033. 27040774 10.1016/j.cub.2016.02.001

[bib19] Fornaciai, M., Brannon, E. M., Woldorff, M. G., & Park, J. (2017). Numerosity processing in early visual cortex. *NeuroImage,* 157, 429–438. 28583882 10.1016/j.neuroimage.2017.05.069 PMC6697050

[bib20] Gallagher, R. M., Suddendorf, T., & Arnold, D. H. (2019). Confidence as a diagnostic tool for perceptual aftereffects. *Scientific Reports,* 9(1), 7124. 31073187 10.1038/s41598-019-43170-1 PMC6509108

[bib21] Gloriani, A. H., & Schütz, A. C. (2019). Humans Trust Central Vision More Than Peripheral Vision Even in the Dark. *Current Biology: CB,* 29(7), 1206–1210.e4. 30905606 10.1016/j.cub.2019.02.023 PMC6453110

[bib22] Hübner, C., & Schütz, A. C. (2017). Numerosity estimation benefits from transsaccadic information integration. *Journal of Vision,* 17(13), 12. 10.1167/17.13.12 PMC5706635 29149766

[bib23] Kanizsa, G. (1979). *Organization in Vision*. New York: Praeger.

[bib24] Knotts, J. D., Odegaard, B., Lau, H., & Rosenthal, D. (2019). Subjective inflation: phenomenology's get-rich-quick scheme. *Current Opinion in Psychology,* 29, 49–55. 30503986 10.1016/j.copsyc.2018.11.006 PMC6517074

[bib25] Komatsu, H. (2006). The neural mechanisms of perceptual filling-in. *Nature Reviews. Neuroscience,* 7(3), 220–231. 16495943 10.1038/nrn1869

[bib26] Lei, Q., & Reeves, A. (2018). When the weaker conquer: A contrast-dependent illusion of visual numerosity. *Journal of Vision,* 18(7), 8. 10.1167/18.7.8 30029271

[bib27] Luna, R., Serrano-Pedraza, I., Gegenfurtner, K. R., Schütz, A. C., & Souto, D. (2021). Achieving visual stability during smooth pursuit eye movements: Directional and confidence judgements favor a recalibration model. *Vision Research,* 184, 58–73. 33873123 10.1016/j.visres.2021.03.003

[bib28] Ma, W. J., Kording, K., & Goldreich. (2022). *Bayesian Models of Perception and Action*. Cambridge, MA: MIT Press.

[bib29] Maldonado Moscoso, P. A., Cicchini, G. M., Arrighi, R., & Burr, D. C. (2020). Adaptation to hand-tapping affects sensory processing of numerosity directly: evidence from reaction times and confidence. *Proceedings. Biological Sciences,* 287(1927), 20200801. 32453983 10.1098/rspb.2020.0801 PMC7287367

[bib30] Mamassian, P. (2016). Visual Confidence. *Annual Review of Vision Science,* 2, 459–481. 10.1146/annurev-vision-111815-114630 28532359

[bib31] Mamassian, P., Landy, M., & Maloney, L. T. (2002). Bayesian modelling of visual perception. In R. P. N. Rao, B. A. Olshausen & M. S. Lewicki (Eds.), *Probabilistic Models of the Brain: Perception and Neural Function,* 13, 13–36.

[bib32] Michotte, A., Thinès, G., & Crabbé, G. (1964). Les complements amodaux des structures perceptives [Amodal completion of perceptial structures]. In G. Thinés, A. Costall, & G. Butterworth (Eds.), *Michotte's experimental phenomenology of perception* (pp. 140–169). Hillsdale, NJ: Erlbaum.

[bib33] Munakata, Y., McClelland, J. L., Johnson, M. H., & Siegler, R. S. (1997). Rethinking infant knowledge: toward an adaptive process account of successes and failures in object permanence tasks. *Psychological Review,* 104(4), 686–713. 9337629 10.1037/0033-295x.104.4.686

[bib34] Nanay, B. (2018). The Importance of Amodal Completion in Everyday Perception. *i-Perception,* 9(4), 2041669518788887. 10.1177/2041669518788887 PMC6083800 30109014

[bib35] Nieder, A., Freedman, D. J., & Miller, E. K. (2002). Representation of the quantity of visual items in the primate prefrontal cortex. *Science (New York, N.Y.),* 297(5587), 1708–1711 12215649 10.1126/science.1072493

[bib36] Odegaard, B., Chang, M. Y., Lau, H., & Cheung, S.-H. (2018). Inflation versus filling-in: why we feel we see more than we actually do in peripheral vision. *Philosophical Transactions of the Royal Society of London. Series B, Biological Sciences,* 373(1755), 20170345. 30061459 10.1098/rstb.2017.0345 PMC6074087

[bib37] Øhrn, H., Svalebjørg, M., Andersen, S., Ring, A. E., & Ekroll, V. (2019). A Perceptual Illusion of Empty Space Can Create a Perceptual Illusion of Levitation. *i-Perception,* 10(6), 2041669519897681. 35186247 10.1177/2041669519897681 PMC8850979

[bib38] Palmer, S. E., Brooks, J. L., & Lai, K. S. (2007). The occlusion illusion: partial modal completion or apparent distance? *Perception,* 36(5), 650–669. 17624113 10.1068/p5694

[bib39] Pessoa, L., Thompson, E., & Noë, A. (1998). Finding out about filling-in: a guide to perceptual completion for visual science and the philosophy of perception. *The Behavioral and Brain Sciences,* 21(6), 723–748; discussion 748-802. 10191878 10.1017/s0140525x98001757

[bib40] Pomè, A., Anobile, G., Cicchini, G. M., & Burr, D. C. (2019). Different reaction-times for subitizing, estimation, and texture. *Journal of Vision,* 19(6), 14. 10.1167/19.6.14 31194220

[bib41] Ramachandran, V. S. (1992). Blind spots. *Scientific American,* 266(5), 86–91. 10.1038/scientificamerican0592-86 1566041

[bib42] Recommendation ITU-R BT.601. Studio encoding parameters of digital television for standard 4: 3 and wide-screen 16: 9 aspect ratios. *International. Radio Consultative Committee International Telecommunication Union*. Retrieved from https://www.itu.int/rec/R-REC-BT.601/.

[bib43] Roitman, J. D., Brannon, E. M., & Platt, M. L. (2007). Monotonic coding of numerosity in macaque lateral intraparietal area. *PLoS Biology,* 5(8), e208. 17676978 10.1371/journal.pbio.0050208 PMC1925133

[bib44] Rosenholtz, R. (2016). Capabilities and Limitations of Peripheral Vision. *Annual Review of Vision Science,* 2, 437–457. 10.1146/annurev-vision-082114-035733 28532349

[bib45] Scaccia, M., & Langer, M. S. (2019). Density discrimination with occlusions in 3D clutter. *Journal of Vision,* 19(12), 10. 10.1167/19.12.10 31621816

[bib46] Scherzer, T. R., & Ekroll, V. (2015). Partial modal completion under occlusion: what do modal and amodal percepts represent? *Journal of Vision,* 15(1), 15.1.22. 10.1167/15.1.22 25613760

[bib47] Scherzer, T. R., & Faul, F. (2019). From Michotte Until Today: Why the Dichotomous Classification of Modal and Amodal Completions Is Inadequate. *i-Perception,* 10(3), 2041669519841639. 31205667 10.1177/2041669519841639 PMC6537272

[bib48] Schütz, A. C. (2012). There's more behind it: perceived depth order biases perceived numerosity/density. *Journal of Vision,* 12(12), 9. 10.1167/12.12.9 23151411

[bib49] Schwarz, G. (1978). Estimating the Dimension of a Model. *The Annals of Statistics,* 6(2), 461–464.

[bib50] Smith, A. T., Singh, K. D., Williams, A. L., & Greenlee, M. W. (2001). Estimating receptive field size from fMRI data in human striate and extrastriate visual cortex. *Cerebral Cortex (New York, N.Y.: 1991),* 11(12), 1182–1190. 11709489 10.1093/cercor/11.12.1182

[bib51] Solovey, G., Graney, G. G., & Lau, H. (2015). A decisional account of subjective inflation of visual perception at the periphery. *Attention, Perception & Psychophysics,* 77(1), 258–271. 10.3758/s13414-014-0769-1 25248620

[bib52] Stewart, E. E. M., Valsecchi, M., & Schütz, A. C. (2020). A review of interactions between peripheral and foveal vision. *Journal of Vision,* 20(12), 2. 10.1167/jov.20.12.2 PMC7645222 33141171

[bib53] Strasburger, H., Rentschler, I., & Jüttner, M. (2011). Peripheral vision and pattern recognition: a review. *Journal of Vision,* 11(5), 13. 10.1167/11.5.13 PMC11073400 22207654

[bib54] Testolin, A., & McClelland, J. L. (2021). Do estimates of numerosity really adhere to Weber's law? A reexamination of two case studies. *Psychonomic Bulletin & Review,* 28(1), 158–168. 32949010 10.3758/s13423-020-01801-z PMC7870758

[bib55] Thielen, J., Bosch, S. E., van Leeuwen, T. M., van Gerven, M. A. J., & van Lier, R. (2019). Neuroimaging Findings on Amodal Completion: A Review. *i-Perception,* 10(2), 2041669519840047. 31007887 10.1177/2041669519840047 PMC6457032

[bib56] Valsecchi, M., Toscani, M., & Gegenfurtner, K. R. (2013). Perceived numerosity is reduced in peripheral vision. *Journal of Vision,* 13(13), 7. 10.1167/13.13.7 24198398

[bib57] van Lier, R. J., van der Helm, P. A., & Leeuwenberg, E. L. J. (1995). Competing global and local completions in visual occlusion. *Journal of Experimental Psychology: Human Perception and Performance,* 21(3), 571–583. 7790834 10.1037//0096-1523.21.3.571

[bib58] Webster, J., Kay, P., & Webster, M. A. (2014). Perceiving the average hue of color arrays. *Journal of the Optical Society of America. A, Optics, Image Science, and Vision,* 31(4), A283–A292. 24695184 10.1364/JOSAA.31.00A283 PMC3979548

[bib59] Weil, R. S., & Rees, G. (2011). A new taxonomy for perceptual filling-in. *Brain Research Reviews,* 67(1-2), 40–55. 21059374 10.1016/j.brainresrev.2010.10.004 PMC3119792

[bib60] Whitney, D., & Yamanashi Leib, A. (2018). Ensemble Perception. *Annual Review of Psychology,* 69, 105–129. 10.1146/annurev-psych-010416-044232 28892638

